# Supplementary material for: Universality in long-distance geometry and quantum complexity
Source: Nature. 2023 Oct 4;622(7981):58–62. doi: 10.1038/s41586-023-06460-3 (PMC10550822; doi:10.1038/s41586-023-06460-3)
Supplement: Supplementary file 1 — Supplementary Information [file 41586_2023_6460_MOESM1_ESM.pdf]

nature portfolio

https://doi.org/10.1038/s41586-023-06460-3

Supplementary information

Universality in long-distance geometry and quantum complexity

In the format provided by the authors and unedited

Supplementary Information for
Universality in Long-Distance Geometry and Quantum Complexity

Adam R. Brown, Michael H. Freedman, Henry W. Lin, Leonard Susskind

1 Zooming in and zooming out

In this appendix, building on the Connection to Coarse Geometry section in the Methods, we describe a mathematical program to characterize the equivalence classes of metrics on right-invariant Lie groups under a certain equivalence relation. The equivalence relation should be loosely thought of as "zooming out" and only seeing the "coarse" features of the geometry. A possible definition will be presented in Eq. S.2, but first we review some related ideas in the mathematics literature.

As long as Riemannian manifolds have been studied,[1] the idea of fixing a point x and zooming in, that is, multiplying all distances in the ball of radius r, B_r, by $1/r$, $r \ll 1$, has been central. The limit of such rescaling is the tangent space T_x at x, a linear space with a metric.[2] In this paper we consider families of metrics where certain tangent directions are increasingly penalized, that is, are declared longer and longer. In the limit where motion in some direction is not just penalized but forbidden, one arrives at *sub-Riemannian* geometry. A fruitful observation of Gromov [1] is that zooming into a point on a sub-Riemannian manifold[3] endows the tangent space with the structure of a *self-similar nilpotent Lie group.* (The simplest example is the Heisenberg group, which has $[X, Y] = Z$, $[X, Z] = 0 = [Y, Z]$, with Z rescaling as l^2 when X and Y are rescaled[4] by l.) On the other hand we may instead zoom out by considering $r \gg 1$. Such limits[5] of zooming

[1] Going back to the 19th century work of Gauss and Riemann.

[2] That is, a nonsingular inner product.

[3] The finite norm directions are assumed to generate all of T_x under bracket, and an additional uniformity condition is assumed, which is always satisfied by right-invariant metrics on a Lie group.

[4] More generally, the Ball-Box theorem describes how ϵ-balls in self-similar Lie groups are approximated by Riemannian boxes with $O(\epsilon^{k_i})$ side lengths where the integers $\{k_i\}$ are governed by commutator depth.

[5] In the physics literature, this is sometimes referred to as Wigner group contraction [2]. For example,

out are called *asymptotic cones.* Simply connected nilpotent Lie groups G and their co-compact lattices are well-studied in this regard. The details of exactly how quickly the asymptote is approached, and how its geometry compares with the original Lie group geometry, has been an active area of research for 25 years (see [3]).[6]

We found, at least for the low-dimensional cases we could solve exactly, that the computed convergence is actually much faster than the proven estimates[7] (Prop. 3 and Thm. 4 of [3]). We find only a small[8] additive error separates the approximate from the limit. We do not know the scope in which such additive estimates hold, but they have inspired us to believe that in the case of interest to us, $\mathrm{U}(2^N)$, the large-scale behavior of at least a large class of right-invariant metrics may be characterized by broad phases with nearly constant large-scale geometry separated by sharp phase transitions. Let us at least describe a conjectural picture here, and also its conjectural relation to renormalization.

Before describing the picture we need to address the compactness of $\mathrm{U}(2^N)$. Zooming in is always possible. Traditionally, zooming out (choose $r \gg 1$) is restricted to non-compact spaces. This may be summarized by saying "all compact spaces have the coarse geometry of a point". The most famous of all "zoom out" theorems is Gromov's Theorem [4,5] that a finitely generated group of polynomial growth contains a nilpotent subgroup of finite index. Remarkably, this theorem has a beautiful quantitative version [6] applicable to *finite groups*[9]. Since it will be established in [8] that $\mathrm{U}(2^N)$, for many penalty schedules, has diameter exponential in N, the finitary

---

SE(2) is a Wigner contraction of SU(2): keep $\mathcal{I}_x = 1$, and take $\mathcal{I}_y$ & $\mathcal{I}_z$ infinite at fixed $\mathcal{I} \equiv \mathcal{I}_z/\mathcal{I}_y$.

[6]To understand zooming in and out the following pair of examples is instructive: If we start with the Euclidean plane both the zoom in and zoom out are again a Euclidean plane (the tangent space at a point and the asymptotic cone at that point respectively). If we start with the Hyperbolic plane and zoom in to a point we get its tangent space (as expected), if we zoom out we get what is called an R-tree, and infinitely branching dendritic object of topological dimension 1 which branches at a dense subset.

[7]The context of these estimates is again nilpotent Lie groups, but its relevance to metrics on $\mathrm{U}(2^N)$ is discussed in the next paragraph.

[8]For the SU(2) example with a penalty $\mathcal{I}$, Eq. 4 of the main text found that the additive error is $\mathrm{O}(\mathcal{I}^{-1/2})$.

[9]See also [7] for some discussion of coarse geometry in the context of a finite group model of complexity geometry.

philosophy (essentially controlled rescaling) can be applied to think about intermediate- and large-scale geometry of this very large, albeit compact, space.

We interpret the distance function on $\mathrm{U}(2^N)$, which depends on a choice of right-invariant[10] metric g and amounts to defining the distance from $\mathbb{1}$, as the complexity. Equation 10 from the Methods is equivalent to the statement that this metric is given by

$$\mathrm{dist}_g(U) = \mathrm{inf}_\gamma \int_0^1 ds \langle \dot{\gamma}, \dot{\gamma} \rangle_g^{1/2}, \tag{S.1}$$

where the infimum is over piece-wise smooth paths from $\mathbb{1}$ to U. The same formula applies to the sub-Riemannian case, $\mathcal{I}_k \to \infty$. (We remind the reader that $\mathcal{I}_k$ are the diagonal elements of the metric on Pauli words of weight k, see Eq. 10 of the Methods.)

Since zooming out tends to blow up curvatures, even if one starts with a Riemannian metric, one may expect a sub-Riemannian (or worse) asymptotic cone in the zoom-out limit. Returning to the Heisenberg group, rescaling a right invariant metric, will, in the limit result in a Carathéodory geometry. Slightly more generally, much has been understood regarding scaling limits of nilpotent Lie groups, [3]: 1. How these rescaled spaces approach their limits, (see Thm 2 of [3]), 2. What is required for two different geometries to have the same limit, (see Prop 3 of [3]), and 3. How finitely generated subgroups approximate the continuous geometry (see Thm 4 of [3]). We feel all three discussions are relevant to our program of coarsely classifying right-invariant metrics on $\mathrm{U}(2^N)$.

In the cited results, the general pattern is that large-scale behavior is controlled to some tolerance, a power law intermediate between an constant additive and constant multiplicative error, by the behavior of the metric when reduced to the Abelianization $\mathfrak{g}/[\mathfrak{g}, \mathfrak{g}]$ of the group's Lie algebra.

The analogy we see is that our "easy" directions (in $\mathfrak{u}(2^N)$), being bracket complete, play the role of $\mathfrak{g}/[\mathfrak{g}, \mathfrak{g}]$. We expect metrics which agree on easy directions will have very similar intermediate and large-scale properties, corresponding to 2 above. Points 1 and 3 also play a role in the

[10] In the mathematics literature it is common to talk about left-invariant (but not necessarily right-invariant) metrics, but here we follow the (mirror-image) physics convention of considering right-invariant (but not necessarily left-invariant) metrics.

analogy. Comparison of Riemannian spaces to their sub-Riemannian limits should be a proof technique for extending 2 from nilpotent Lie groups to $U(2^N)$. But the translation will require ideas such as in [41] because $U(2^N)$ is compact, so the "approach to infinity" must be carefully quantified. Point 3 should help us make connections between the gate-based, and differential-geometric methods of navigating $U(2^N)$.

Here is a goal for making the analogy concrete. Give a criterion capable, in interesting cases, of splitting the spectrum to identifying the "easy" subspace $E \subset \mathfrak{u}(2^N)$. Then g_0 is defined to be the sub-Riemannian metric obtained by simply retaining the metric on E and forbidding motion in other directions. If E is *effectively* bracket complete (effective in the sense that the completion is achieved with reasonable numerical efficiency), then we would like to establish the following conjectural inequality:

$$|\mathrm{dist}_g(U_1, U_2) - \mathrm{dist}_{g_0}(U_1, U_2)| \leq \mathrm{O}((\mathrm{dist}_g(U_1, U_2))^{1-\alpha}) + C_{g_0} \qquad \text{(S.2)}$$

for $U_1, U_2 \in U(2^N)$, some $\alpha \in (0, 1)$, and $C_{g_0} > 0$ is a constant which is order $O(1)$ when E efficiently generates $\mathfrak{g}$ under brackets.

For large scales, comparable to the diameter, the most optimistic conjecture is that distances induced by the sub-Riemannian metric g_0 are *additively* close to those of an approximating Riemannian metric g. We require, of course, that g agree with g_0 on the bracket generating subspace E (for example 1- and 2-qubit Pauli words) and that the penalties defining g in orthogonal directions are reasonably large.[11] We do not have in hand technical tools powerful enough to attempt a proof. Rather than sketching a proof, contrariwise, we will explain a few fundamental obstacles to finding one.

The perturbative calculations in [9] seem to have reached their natural limit and fresh ideas are needed. As discussed in the Methods, in [8] it will be shown that metrics with a sufficiently rapidly growing penalty schedule have diameters exponential in N. It will further be shown [9] that a U with distance d from $\mathbb{1}$ in a sufficiently expensive penalty metric can be ϵ-approached by a path of length $\epsilon^{-1}d^2$ in the sub-Riemannian cliff metric, see Eq. 18 of the main text. Here "ϵ-approached" means the path arrives within a Killing-metric ball of radius ϵ. However, the ball-box theorem warns us

[11] This is the sense in which g *approximates* g_0, where the normal penalties are infinite.

that ϵ-approach may still leave us a tiny power of ϵ, like $\epsilon^{0.001}$, away in the metrics of interest. This phenomenon is well-known for nilpotent groups[12] [3] where it is captured by the notion of *abnormal geodesic arc.* This is a geodesic arc in the sub-Riemannian geometry whose ϵ-variations fail to cover any Riemannian $O(\epsilon)$ balls around its endpoints. So, notoriously, it is hard to solve your problems at the last moment by first arriving Killing-close and then proceeding to the desired target. This has led us to ask if there is a *lever arm* here to exploit: If we recognize, far off, that we will slightly (in the Killing norm) miss our target, can we make inexpensive adjustments early (a strategy we called 'wiggle room')? If possible, learning how to do this seems tantamount to proving at least a weakened version of our conjecture, where even beyond a multiplicative constant we would allow a power-law expansion of distance.

Actually, Ref. [10] provides further evidence that the critical/cliff correspondence pertains not only at the largest scale (the diameter) but also at intermediate scales. This is addressed by comparing covering numbers $c_{\text{critical}}(r)$ and $c_{\text{cliff}}(r)$, the number of r-balls required to cover the manifold. For $r =$ diameter, this number is 1; as $r \to 0$, it is a surrogate for volume. As r varies, the covering number probes the geometry on a range of scales (see the discussion of marbles in the main text[13]).

So for the case where only 2-qubit interactions are easy, our conjectural picture is that there is some *critical* metric g_{crit} determined by a critical schedule of penalties $\bar{\mathcal{I}}_k$, with $\mathcal{I}_1$ and $\mathcal{I}_2$ fixed, which lies at the *lower* edge of the phase of metrics which at its upper limit is the metric with $\mathcal{I}_{k\geq 3} = \infty$, the sub-Riemannian metric. Thus little should happen to already large distances when penalties $\mathcal{I}_{k\geq 3}$ are increased but if any $\mathcal{I}_k$ are reduced certain large distances shrink at first order.

It is interesting to ask: is $\bar{\mathcal{I}}_k$ unique (or perhaps forming a convex set?). If it is unique, perhaps it admits several other characterizations. Also, what kind of renormalization group flow might take us toward $\bar{\mathcal{I}}_k$?

---

[12] Nilpotent groups are relevant to us both in the small where they arise [6] as a structure on the tangent space of a sub-Riemannian manifold, and conjecturally in the zoom-out limit, provided approximate asymptotic cones can be defined.

[13] Since in flat T^d the number of marbles of radius n we can cram in scales like $n(r) \sim r^{-d}$, the $n(r) \sim r^{-4}$ scaling we saw at some scales for the 'three-dimensional' Berger sphere is characteristic of four dimensions (see e.g. [11]), and so exhibits what in quantum field theory is called an 'anomalous dimension'.

Among metrics with a prescribed fixed value on 1- and 2-qubit Hamiltonians, let us speculate on possible characterizations of $\bar{\mathcal{I}}_k$. A first possibility is that using the natural partial order on norms, $\bar{\mathcal{I}}_k$ should be an infimum penalty schedule among those where the first variation of diameter vanishes, $\frac{\partial(\text{diameter})}{\partial g} = 0$ (when the variation is presumed constant on the space spanned by 1- and 2-qubit Hamiltonians), and a supremum metric among all such metrics with $\frac{\partial(\text{diameter})}{\partial g} \neq 0$. Another possibility is to use the spectrum of the Laplacian, e.g., λ_1 the first non-zero eigenvalue of ∇^2 on the group manifold. Motivated by [12] where overcompleteness drives up a lowerbound for λ_1, the critical metric (from a very broad class—perhaps even including Finsler metrics, e.g. taxicab metrics) may be the one maximizing $\lambda_1(\text{diameter})^2$, again constrained on 1- and 2-qubit Hamiltonians. Furthermore, the collapse of sectional curvatures, $|K|$, in the qubit context near the (perhaps critical) exponential metric can be seen as reinforcing this conjecture. For example, among all geometries on the 2-sphere with diameter π, λ_1 is apparently maximized by the round metric. Relatedly, a probe that is sensitive to parallelism could come from the notion of conductance, if we imagine that the complexity geometry is fabricated from a material of constant resistivity. Perhaps the critical metric maximizes $c(\text{diameter})^{2-4^N}$, where c is the average conductivity between all pairs of points $U_1, U_2 \in \mathrm{U}(2^N)$, constrained as above. This relates to the 'load-balancing' property we expect for the critical metric. A final possibility is that the critical metric could minimize the average distance to the cut locus over the visual sphere, constrained as above.

To complete our conjectural picture, imagine a flow which seeks to lower all $\mathcal{I}_k$, $k \geq 3$, but constantly and homothetically rescales $\{\mathcal{I}_3, \mathcal{I}_4, \dots\}$ to maintain constant diameter. A flow of this type, if initialized in the stable phase near the sub-Riemannian g_0, would be expected to converge to g_{crit}. One can generalize this flow to general right-invariant Riemannian metrics (again fixed on the span L of 1- and 2-qubit Hamiltonians) to a *random* flow. Randomly select an eigen (principal) direction of g in $L^\perp$, shorten it, and then homothetically rescale $g|_{L^\perp}$ to keep the diameter constant. A flow of this type might be considered as a kind of renormalization of right-invariant metrics on $\mathrm{U}(2^N)$. A natural question is whether the flow of geometries from 'UV' to 'IR' has anything to do with Ricci flow, which

has a known connection to the renormalization group of 2D quantum field theories. (See e.g., [13] for a discussion of Ricci flow in the context of homogeneous geometries on group manifolds.) This seems unlikely to us as the conjectured "fixed points" are not Einstein metrics, although perhaps there is a generalization of the concept that could be applicable.

There is also the question: should the flow be defined only on metrics which know about some fixed qubit structure—as for those we have so far discussed—or in the spirit of [14] and [15] should we consider all right-invariant metrics and ask that the flow have fixed points which locate a qubit structure and a critical schedule together.

The large-scale geometry of right-invariant metrics, even for exceedingly simple penalty schedules, is enormously subtle. The successful proofs (e.g. diameter lowerbounds) are all indirect. To highlight how little is known explicitly, without complexity assumptions, there seems to be no explicit unitaries whose sub-Riemannian cliff metric distance from $\mathbb{1}$ is known to grow superlinearly with N; see however [16] for an interesting construction that relies on a complexity assumption about PSPACE.

Another question whose answer is not yet known: at long distances (comparable to the diameter) is there a "high frequency" component to $|\text{dist}_{\text{cliff}} - \text{dist}_{\text{crit}}|$? Essentially this asks if $d_{\text{cliff}}(\mathbb{1}, U) = \ell \sim \text{diam}$ and $d_{\text{crit}}(U, U') = \epsilon > 0$ then is $d_{\text{cliff}}(\mathbb{1}, U') \leq \ell + \mathrm{O}(\epsilon)$? A version of this question was addressed in the context of a specific 5-dimensional nilpotent Lie group [3]. The authors [3] showed that the "cliff metric" on this group has an abnormal geodesic segment, i.e. a geodesic arc of any desired length in some right-invariant sub-Riemannian geometry, whose ϵ variations fail to cover an $\mathrm{O}(\epsilon)$-Riemannian neighborhood of its endpoints. So in that example we would say there is a high-frequency discrepancy at all scales. On $\mathrm{U}(2^N)$ we know of no analogous abnormality and would guess that the very rich dynamics of geodesics should rule it out, but have no proof. Perhaps the simplest reason for expecting a smooth relationship is the lack of any candidate "quantum number."

More generally, beyond the complexity geometry examples on $\mathrm{U}(2^N)$, we may ask for a classification of the equivalence classes of coarse geometries of metrics on non-compact Lie groups, or sequences of compact Lie groups. Such a mathematical program would significantly generalize existing work on the coarse geometry of finitely-generated discrete groups.

2 Holography, load balancing, and the critical metric

In the Methods we described how the conjectures of this paper, if correct, could give a robust theoretical underpinning to the holographic complexity conjectures. In this section, we will describe further implications.

First, let's note that as well as being informed by the conjectures in this paper, the holographic complexity conjecture also in turn informs our expectations for the 'critical metric', defined in the Main Conjectures section of the main text. In the Methods, we described evidence that the binomial metric is in the same universality class as the cliff metric. Could the binomial metric be not only *in* the universality class, but be the cheapest member of the class, i.e., be the critical metric? On the one hand, the binomial metric shares with the exponential metric the property that the sectional curvatures are modest, which we argued is one signature of being the critical metric. On the other hand, if the binomial metric really were the critical metric, an odd consequence would follow from the fact the binomial metric has an N-dependence that changes with k, $\mathcal{I}_k \sim N^{2k}$. If the binomial metric were the critical metric, this would imply that the complexification rate of k-local Hamiltonians is never extensive in N for all values of k, even if we multiply the entire penalty schedule by an overall N-dependent factor. This is different from the expected complexification rate of Brownian circuits, as well as the gate complexification rate, and from the rate we expect of black holes. This is one reason to prefer the exponential metric, given by Eq. 12 of the main text, as a candidate for the critical metric: for the exponential metric, $\mathcal{I}_k$ depends only on k and is independent of N. (Though note also that for generic k-local Hamiltonians with $\text{Tr}[H^2] = 1$ the rate of change of *inner-product* distance is not extensive and instead scales like $\sqrt{N}$, essentially due to Pythagoras' theorem.)

Second, as a subject for further study, recall that we described how the critical metric is approximately load-balanced, meaning that there are many different paths, all with roughly the same complexity, that connect any two distant states. It would be interesting to explore what this degeneracy might mean in the context of bulk physics.

Finally, let's note that one should not confuse the sensitivity of the

complexity to the choice of gates or penalty factors with the sensitivity of the complexity to the choice of UV regulator of the quantum field theory, discussed in [17–20]. For example, the SYK model is UV finite, but one still needs to discuss the choice of penalty factors in the definition of complexity.

3 Two-dimensional example

In the introduction of the main text, we observed the surprising fact that a deformation of the metric that has only a tiny additive effect on the distance between any pair of points can nevertheless drive the volume to infinity. We illustrated this with a three-dimensional homogeneous metric, the Berger sphere. Let's now give an example of this phenomenon in two dimensions. The advantage of the two-dimensional example is that it is easier to visualize; the disadvantage is that we must consider a metric that is inhomogeneous.

The two-dimensional example is the metric described in Sec. 3 of Ref. [21]. While our three-dimensional example in the main text was the *unitary* complexity of a single qubit, given by a deformed three-sphere (a non-bi-invariant metric on SU(2)), our two-dimensional example is the *state* complexity of a single qubit, given by a deformed two-sphere (a deformed Bloch sphere). The distance between two points on the deformed Bloch sphere is defined as the *unitary* complexity of the least complex unitary that connects them, where the unitary complexity is given by Eq. 1 of the main text. This is explained in detail in Ref. [21], along with a geometric visualization. The line element is

$$4\,ds^2 = d\theta^2 + \frac{\mathcal{I}\sin^2\theta}{\mathcal{I}\cos^2\theta + \sin^2\theta}d\phi^2. \tag{S.3}$$

For $\mathcal{I} = 1$ two points are separated by the standard angular distance on a round two-sphere (the inner-product distance). For $\mathcal{I} > 1$ rotations of the sphere are more expensive when the axis of rotation has a component in the vertical direction, σ_z. As $\mathcal{I}$ becomes ginormous, the only rotations that are not prohibitively expensive have rotation axes that are equatorial.

To manifest the surprising phenomenon, we start with $\mathcal{I}$ very large, and then take the limit $\mathcal{I} \to \infty$. On the one hand this makes only a tiny additive change to distances, since (via its definition) state complexity

inherits Eq. 4 from unitary complexity. On the other hand the volume goes (logarithmically) to infinity, since two neighbouring points on the equator have their distance multiplied by a huge factor, $\sqrt{\mathcal{I}}$. The limit of the geometry as $\mathcal{I} \to \infty$, and its relation to the optimal shape for tunnels bored through the Earth, is described in detail in Ref. [21].

References

[1] Mikhael Gromov, "Carnot-Carathéodory spaces seen from within", In *IHÉS publication* 1994, and in *Sub-Riemannian geometry*, pages 79–323, Springer, 1996.

[2] E. Inonu and E. P. Wigner, "On the Contraction of groups and their represenations," Proc. Nat. Acad. Sci. **39**, 510-524 (1953).

[3] Emmanuel Breuillard and Enrico Le Donne. "On the rate of convergence to the asymptotic cone for nilpotent groups and sub-Finsler geometry", *Proc. Natl. Acad. Sci.*, 110(48):19220–19226, 2013.

[4] Gromov, M., Asymptotic invariants of infinite groups, (Inst. Hautes Etud. Sci.,1992), https://cds.cern.ch/record/234998

[5] Mikhael Gromov. "Groups of polynomial growth and expanding maps", *Publ. Math. IHÉS*, 53(1):53–78, 1981.

[6] Yehuda Shalom and Terence Tao, "A finitary version of Gromov's polynomial growth theorem", *Geom. Funct. Anal.*, 20(6):1502–1547, 2010.

[7] H. W. Lin, "Cayley graphs and complexity geometry," JHEP **1902**, 063 (2019) [arXiv:1808.06620 [hep-th]].

[8] A. R. Brown, "A quantum complexity lower bound from differential geometry," Nature Phys. **19**, no.3, 401-406 (2023) [arXiv:2112.05724 [hep-th]].

[9] A. R. Brown, "Polynomial Equivalence of Complexity Geometries," [arXiv:2205.04485 [quant-ph]].

[10] M. Freedman, "Critical Metrics and Covering Number," [arXiv:2205.13638 [quant-ph]].

[11] N. Eldredge, M. Gordina, & L. Saloff-Coste, "Left-invariant geometries on SU(2) are uniformly doubling", Geom. Funct. Anal. **28**, 1321-1367 (2018).

[12] Fan Chung. *"Spectral graph theory"*, Number 92 in CBMS Regional Conference Series in Mathematics. American Mathematical Society, 1997.

[13] Glickenstein, D. & Payne, T. Ricci flow on three-dimensional, unimodular metric Lie algebras, *Communications In Analysis And Geometry*, **18**, 927-961 (2010).

[14] M. Freedman and M. S. Zini, "The Universe from a Single Particle," JHEP **01**, 140 (2021) [arXiv:2011.05917 [hep-th]].

[15] M. Freedman and M. S. Zini, "The universe from a single particle. Part II," JHEP **21**, 102 (2021) [arXiv:2108.12709 [hep-th]].

[16] T. C. Bohdanowicz and F. G. S. L. Brandão, "Universal Hamiltonians for Exponentially Long Simulation," [arXiv:1710.02625 [quant-ph]].

[17] S. Chapman, M. P. Heller, H. Marrochio and F. Pastawski, "Toward a Definition of Complexity for Quantum Field Theory States," Phys. Rev. Lett. **120**, no. 12, 121602 (2018) [arXiv:1707.08582 [hep-th]].

[18] R. Jefferson and R. C. Myers, "Circuit complexity in quantum field theory," JHEP **1710**, 107 (2017) [arXiv:1707.08570 [hep-th]].

[19] R. Q. Yang, "Complexity for quantum field theory states and applications to thermofield double states," Phys. Rev. D **97**, no. 6, 066004 (2018) [arXiv:1709.00921 [hep-th]].

[20] A. Bhattacharyya, A. Shekar and A. Sinha, "Circuit complexity in interacting QFTs and RG flows," JHEP **1810**, 140 (2018) [arXiv:1808.03105 [hep-th]].

[21] A. R. Brown and L. Susskind, “Complexity geometry of a single qubit,” Phys. Rev. D **100**, no. 4, 046020 (2019) [arXiv:1903.12621 [hep-th]].
